# Recycling of metallocene isotactic polypropylene: importance of antioxidants

**DOI:** 10.1007/s10973-022-11505-2

**Published:** 2022-08-12

**Authors:** Enrique Blázquez-Blázquez, Tamara M. Díez-Rodríguez, Ernesto Pérez, María L. Cerrada

**Affiliations:** Instituto de Ciencia y Tecnología de Polímeros (ICTP-CSIC), Madrid, Spain

**Keywords:** Recycling; Antioxidants, Polypropylene, Young’s modulus; Strain at break

## Abstract

Recycling of plastics is absolutely essential in a circular economy, especially in the case of commodity polymers from fossil resources, like isotactic polypropylene (iPP). Therefore, evaluation of the factors that are decisive for an optimum performance of the recycled based materials becomes mandatory for the obtainment of new products with optimal properties. One of the most important aspects is the protection of the plastics materials not only against the external degradation agents, but also from the radicals generated during their previous service life. Accordingly, several materials have been prepared by extrusion based on virgin iPP with different amounts of the same polypropylene severely degraded, which has been used as model component to be recycled. Previous to the extrusion, a mixture of antioxidants was added to all the samples, and special attention has been paid to consumption of those additives during the extrusion. The results show an increasing reduction of antioxidants with rising content of the degraded material. But, importantly, a rather analogous mechanical response has been found for all the recycled materials in relation to the virgin iPP, pointing out a satisfactory dilution effect of the existing degradation points within the virgin polymeric chains, and indicating the very relevant action of the antioxidants used.

## Introduction

The massive consumption of plastics, mainly in applications of a unique use, has turned out in an enormous amount of plastic wastes. Isotactic polypropylene (iPP) is extensively employed in packaging, consumer products and automotive industry owing to its excellent mechanical, thermal and gas permeability properties together with its good processability and relatively low cost. Its massive use makes it one of the main fractions in the aforementioned plastic wastes, currently worsened by the COVID-19 pandemic due to the huge amount of polypropylene masks employed every day worldwide, together with the rest of single-use protection textiles and sanitary material [1].

Interest in the plastics recycling has exponentially increased in the last two decades as a result of the importance given to provide successive uses to these plastics and also to reduce the consumption of raw materials from fossil resources. At governmental level, many strategies have been lately implemented to promote transition in the global economy from linear to a circular model and more specifically in the field of plastics. Consequently, deployment of recycled plastics for new objects and products has been boosted [2, 3]. An optimization of recycling techniques must occur in order to contribute to the development of more efficient processes, concerning stakeholder commitments across the polymer manufacturing industry [4, 5]. Currently, the sustainable management of plastics mainly involves reusing, mechanical recycling and chemical recycling [6], although the protocols require to be improved.

A common strategy used in plastics materials for achievement of others with equivalent properties involves preparation of mixtures between recycled and virgin polymers. This approach has been used for isotactic polypropylene (iPP) [7, 8], where employment of recycled iPP (iPPr) turns out very interesting because its huge number of applications. Therefore, one of the main goals to be attained during recycling is the obtainment of high quality materials from post-consumer and post-industrial waste streams to use them in the manufacturing of new products, as compost bins, construction materials, or extruded and injection-molded parts for automotive industry, among others [9–11].

Mechanical recycling, especially in polyolefins, shows certain limitations, being the oxidative polymer degradation one of the main negative aspects to be treated since it can significantly reduce the resultant thermal and mechanical properties, as well as the final aesthetic quality of the future materials. Thus, mechanically recyclable plastics might have undergone detrimental pre-oxidation, chain scission or cross-linking, containing also residual additives from their previous shelf life. All these drawbacks could be controlled, in most cases, by a further incorporation of stabilizers [12–14]. It is well established that the use of additives can tune different properties, performances and stability for raw plastics. However, this stabilization strategy has been proved less effective for recycled polymers with a previous service life [15–17], so that recycling of plastics turns their further stabilization into a challenge. Accordingly, attention should be paid to interaction phenomena between the radicals existing in polymeric materials and the stabilizing agents added in order to achieve tailored solutions.

Metallocene catalysts employed during the polymerization stage allow obtaining iPP with narrow distributions of molar masses (values of polydispersity close to two) and with a more controlled microstructure than those achieved when conventional Ziegler–Natta catalytic systems are used. Furthermore, there are also variations between both kinds of PPs in type and distribution along their macrochains of the insertion mistakes (stereodefects and regiodefects), these defects affecting the average length of the crystallizable (fully isotactic) sequences. Therefore, their crystalline characteristics (mainly type of polymorphs to be developed, degree of crystallinity and crystal size) are somehow different. Since the most of the investigations related to iPP degradation and recycling capacity are performed on Ziegler–Natta iPP, evaluation of metallocene iPPs turns out very interesting in order to gain knowledge on their behavior under these conditions and to find out dissimilarities or analogies between them. Accordingly, characteristics of a metallocene iPP after being subjected to a severe thermo-oxidation process were described in a previous study [13]. Some of those materials showed an advanced state of degradation due to their exposure to extreme conditions, which led to an important reduction in their properties. A noticeable presence of carbonyl species was observed through ATR-FTIR together with a considerable delay in crystallization and inhibition of formation of the γ polymorph, all these features being associated with the advance of degradation. Consumption of antioxidants initiated this decomposition, indicating the importance of their presence in the final durability of the polymeric materials.

Thus, the aim of this study is to analyze an antioxidant mixture, based on common compounds used for iPP, and in sufficient high amounts for obtaining recycled materials by melt extrusion with optimal ultimate performances. The strategy followed in this research constitutes a novelty compared to other studies previously described since, on one hand, the iPP analyzed has been synthesized with a metalocene catalyst instead of with the more common Ziegler–Natta ones. And, on the other hand, consumption of the added antioxidants is evaluated, the dependence of their depletion as function of their nature, as well as the influence that their remaining amount and the content in a severely degraded iPP play in the phase transitions and in the crystalline structure. Performances of the different materials have been checked at room temperature and also at high temperatures. Thus, these recycled plastics will be prepared from combination of several contents of an importantly degraded iPP with the same virgin iPP. Then, goodness of the mixture of antioxidants chosen will be analyzed from its consumption along the processing of those recycled materials, and from their influence on the global structural, thermal and mechanical characteristics. Accordingly, these recycled materials will be examined by melt flow rate, infrared spectroscopy with Fourier Transform (FTIR), oxidation induction time and uniaxial tensile stress–strain measurements. Moreover, changes in crystalline features and in phase transitions will be assessed by differential scanning calorimetry (DSC) and real-time variable-temperature Wide Angle X-ray Diffraction (WAXD) synchrotron experiments. Finally, an analogous thermo-oxidative treatment to that applied for the achievement of the severely degraded iPP has been imposed to these recycled materials in order to estimate the effect of the selected additives in their lifetime.

## Materials and methods

A commercially available metallocene isotactic polypropylene (Metocene HM562P: nominal melt flow index of 15 g/10 min, ISO 1133, kindly supplied as pellets by Lyondell Basell) was selected in this work as polymeric material. It has been designated simply as HM.

The severely degraded iPP to be incorporated into the virgin HM iPP as recycling component was obtained by its thermal-oxidative degradation in a convection oven at 95 °C for 8 days, as established previously [13]. It has been labeled as HMDeg.

Final recycled materials, with different amounts of raw iPP plus severely degraded iPP, were prepared by melt extrusion in a co-rotating twin-screw microextruder Rondol (Microlab model) with a length-to-diameter ratio 20:1. A screw temperature profile of 115, 170, 180, 185 and 190 °C was employed from the hopper to the die. Then, films with a thickness of around 200 μm were processed by compression molding at 190 °C and at 25 bar for 3 min in a hot-plate Collin press (200 × 200 model). A relatively fast cooling, at a rate of around 80 °C/min, was applied from the melt to room temperature between plates under pressure (25 bar). This thermal treatment is rather similar to those applied in the industrial processing.

Previous to the extrusion, a mixture of antioxidants was added to all the samples, which involved 750 ppm of Irgafos 168 (CAS 31570-04-4) and 300 ppm of Irganox 1076 (CAS 2082-79-3). This selection was performed based on the low additive content found in the films processed from virgin iPP pellets, which consisted of 290 ppm in Irgafos 168 [13]. Materials composition, indicating the amount incorporated of severely degraded iPP (HMDeg) and antioxidants, as well as the sample designation, are detailed in Table 1. HMRec100 is, therefore, the severely degraded HMDeg to which the two antioxidants have been incorporated (750 ppm of Irgafos 168 and 300 ppm of Irganox 1076).

**Table 1:** Composition in virgin iPP and in severely degraded iPP (labeled as HM and HMDeg, respectively) together with content in antioxidants and values of MFR for the distinct samples under study

Sample	Virgin HM/mass%	HMDeg/mass%	Irgafos 168/ppm	Irganox 1076/ppm	MFR/g (10 min)^−1^
HMRec0	100	0	750	300	15
HMRec10	90	10	750	300	21
HMRec20	80	20	750	300	31
HMRec40	60	40	750	300	42
HMRec100	0	100	750	300	59

Melt flow rate (MFR) was measured at 230 °C under a load of 2.16 kg according to ISO 1133 norm. Results are listed in Table 1.

Determination of amount of the existing antioxidants after the extrusion processing required a Soxhlet extraction with dichloromethane for 8 h. The extraction solution was concentrated in a rotary evaporator. The obtained residue was transferred to a chromatographic vial and dried with a nitrogen flow.

Analytical determination of antioxidants was carried out using a Hewlett Packard 6890 HRGC gas chromatograph equipped with an Agilent Technologies mass spectrometry detector model 5973. The separation of the compounds was performed on a DB5-HT capillary column (15 m × 250 μm and 0.1 μm). The carrier gas used was helium with a flow rate of 1 mL min^−1^. The electronic impact (70 eV) was the type of ionization selected for the mass spectrometer. The chromatographic protocol was chosen according to previous investigations [13, 18].

Fourier transform infrared spectroscopy using a total attenuated reflectance device (FTIR-ATR) was employed to visualize oxidative species. Spectra were recorded on a PerkinElmer Spectrum Two spectrophotometer with 32 scans and a resolution of 4 cm^−1^.

Calorimetric analyses were performed in a TA Instruments Q100 calorimeter connected to a cooling system and calibrated with different standards. The sample masses were around 7 mg. A temperature interval from − 65 to 200 °C was studied at a scanning rate of 20 °C^.^min^−1^. The first melting and the crystallization processes were evaluated in detail. For the determination of the crystallinity, a value of 160 J g^−1^ [19, 20] was used as the enthalpy of fusion of a perfectly crystalline material.

Real-time variable-temperature WAXD experiments were carried out with synchrotron radiation in beamline BL11-NCD-SWEET at ALBA (Cerdanyola del Valles, Barcelona, Spain) at a fixed wavelength of 0.1 nm. A Rayonix detector has been used at a distance about 19 cm from sample and a tilt angle of around 30 degrees. A Linkam Unit, connected to a cooling system of liquid nitrogen, was employed for the temperature control. The calibration of spacings was obtained by means of silver behenate and Cr_2_O_3_ standards. The initial 2D X-ray images were converted into 1D diffractograms, as function of the scattering vector, *s* = *d*^−1^ = 2 sin *θ*λ^−1^. Film samples of around 5 × 5 × 0.2 mm were used in the synchrotron analysis.

Mechanical behavior of the samples was analyzed from nominal stress–strain tests performed at a temperature of 25 °C and a stretching rate of 10 mm^.^min^−1^ in a MTS *Q*-Test Elite dynamometer with a load-cell of 100 N. Specimens for these experiments were punched out from the polymer films using a dumbbell die. Dimensions of these strips were 15 mm long, 1.9 mm wide and around 0.20 mm thick. At least, six different strips were stretched until fracture for a given specimen.

Oxidation induction time (OIT) was analyzed in a Mettler Toledo DSC822e differential scanning calorimeter (DSC). Samples of about 5 mg were deposited in open aluminum pans, and heated to the test temperature (180 °C) at a rate of 20 °C^.^min^−1^ under a nitrogen atmosphere and maintained at that temperature for 3 min before oxidation began. Then, purge gas was commuted to oxygen and dependence of the heat flow was recorded on time, starting from the switch to an oxidant atmosphere. The OIT value was estimated from time of the oxidation process onset.

Thermo-oxidative experiments were carried out on the different films in a convection oven at 95 °C to determine the durability of the recycled materials. The holding time at this high temperature for each material was estimated from beginning of the whitening of films, as well as from the extreme brittleness exhibited by them. To further corroborate that the degradation in a given material had really started, a DSC cooling test was carried out since a shift to lower values of the crystallization temperature, T_c_, has been described [13] in samples thermally treated at 95 °C, as indicative of a process of severe degradation.

## Results and discussion

**Fig. 1:**
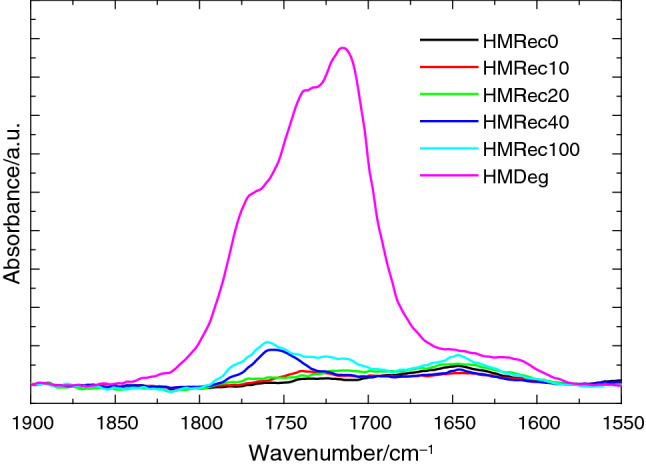
ATR-FTIR spectra in the carbonyl region for the different films evaluated

Figure 1 shows the ATR-FTIR spectra in the carbonyl region for the recycled extruded samples under analysis attained adding different amounts of the severely degraded HMDeg (see Table 1). For a suitable comparison, spectrum of this HMDeg just after its treatment in the oven at 95 °C without addition of the antioxidant mixture is also included. Results clearly indicate the very important role that the supplementary incorporation of antioxidants play, since intensity of carbonyl bands in the severely degraded HMDeg after incorporating additives and its further extrusion (sample HMRec100) is much smaller than that observed in the carbonyl region from the original highly degraded HMDeg used as recycled component. In these ATR measurements, wave could penetrate just some microns into the sample, although these results fully agree with those derived from experiments performed under transmission for these two specimens.

Main degradation mechanisms would occur preferentially on the surface of the HMDeg material, which is in direct contact with the oven air. Furthermore, this oxygen-induced degradation will start in the amorphous regions, since iPP is semicrystalline and difficulty of oxygen to pass through the crystals has been previously reported [21]. The degraded polymeric chains existing in HMDeg will be blended with those raw macrochains from the virgin iPP during the melt extrusion and a dilution effect will occur, causing an overall distribution of degradation nuclei throughout the resultant recycled material. This dilution, together with the neutralization of those oxidative radicals because of the additional incorporation of antioxidants, leads to a significant reduction of the carbonyl species observed in this spectral IR region, as seen in Fig. 1. Moreover, a little increase is also noticeable in the intensity of this carbonyl zone as content in HMDeg is raised. A change is also feasible in the type and ratio of the oxidative species present in these recycled materials, as deduced from the different shape of their spectra when compared with that exhibited by HMDeg. Presence of degraded polymeric chains can be also presumed from the rise observed in the values of MFR as the HMDeg amount is increased in the recycling materials. This is a common effect in other recycled composites [11].

The next aspect to be explored is determination of the amount of antioxidants remaining after the extrusion process for the different recycled HMRec materials, to learn if the initial mixture added, consisting in 750 ppm of Irgafos 168 and 300 ppm of Irganox 1076, behaves similarly in all of them or if there is a dependence in the antioxidant consumption on the initial composition in HMDeg. It has to be recalled that the virgin iPP under study only contained Irgafos 168 in a much lower amount [13].

**Fig. 2:**
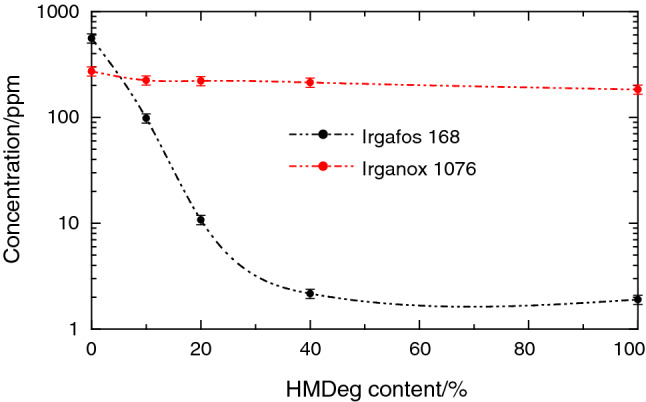
Antioxidants content after extrusion found in the different recycled HMRec materials as a function of their composition in HMDeg, the severely degraded component

Figure 2 shows the antioxidants content remaining in the different samples after extrusion as function of the ratio in severely degraded HMDeg incorporated. Antioxidants consumption is clearly dependent, on one hand, on the HMDeg content and, on the other hand, on nature of the antioxidant. Concerning the former, antioxidants content decreases with increasing amount of HMDeg in the recycled material, which is expected because of the higher fraction in oxidative radicals. And, regarding the latest, it is noticeable that Irgafos 168 is used faster and in a larger amount than Irganox 1076. Irgafos 168 is a hydrolytically stable organophosphite processing stabilizer [22] and, as a secondary antioxidant, it reacts with the hydroperoxides formed along processing by auto-oxidation of polymeric chains. Thus, its presence prevents the processes inducing degradation and allows extending the performance of other primary antioxidants.

Reduction shown in Irganox 1076 is much smaller than that observed for Irgafos 168 and only a small fraction has been consumed along processing by action of the heat and also of the strain imposed by screws through melt extrusion. Therefore, Irganox 1076 contributes to stabilization of the different HMRec materials during their processing, as other primary phenolic antioxidants, and provides long-term thermal stability against thermal degradation [23–25].

Thus, although the same amount of antioxidants was initially incorporated to the different recycled HMRec materials, independently of the content in HMDeg, an evident dissimilarity in their consumption is observed. In order to understand this fact, the mass achieved during the extraction process in soxhlet, from where antioxidants content was assessed, has been further evaluated. In polyolefins, presence of oxygen during their degradation causes free radicals in the chains that can give rise to a wide variety of degradative secondary reactions. Oxygen is fixed to susceptible carbons in the macrochain and peroxides are formed, decomposing next into ketones or aldehydes [26, 27]. Shorter polymeric chains are then generated during oxidative processes, which can be dissolved during the extraction protocol in soxhlet. Consequently, the extract for determination of antioxidants is enriched in this type of degradation products. Its characterization is not possible by GC/MS since their molar masses are out of the working range of this technique. Nevertheless, this extract can be concentrated (to a final volume of 10 μL) and analyzed by infrared spectroscopy due to the capability of this technique for identifying functional groups in polymeric architectures [28, 29].

**Fig. 3:**
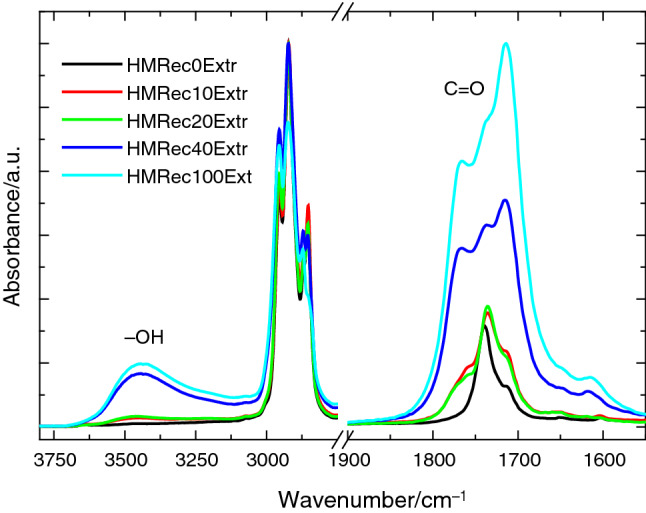
ATR-FTIR spectra of concentrated extracts attained from soxhlet protocol for the different recycled HMRec materials

Figure 3 displays the spectra obtained from these concentrated extracts for all of the recycled HMRec samples, which have been normalized considering the 2950 cm^−1^ band as a reference. The main differences are found in two distinct regions, which correspond to 3100–3500 cm^−1^ and the carbonyl zone, respectively. In both of them, an evident increase with growing content in HMDeg is noticed in the bands associated with polar species, which were caused by degradation. The first region, ranged from 3100 to 3500 cm^−1^, is ascribed to presence of hydroxyl groups from hydroperoxides. Thus, it is associated with the main primary products in the oxidation of polyethylene and other polyolefins [30]. Its appearance is even detected in the extracts from those recycled materials containing the two lowest HMDeg contents: HMRec10extract and HMRec20extract. Its intensity remarkably rises at higher compositions.

The second sensitive spectral zone corresponds to the carbonyl region. Formation of carboxyl or carbonyl groups, associated with the incorporation of oxygen to the hydrocarbon chain, is again noticeably raised when the recycled HMRec materials increase their content in HMDeg, the severely degraded iPP. Signals of carboxyl or carbonyl species from this HMDeg, which shows an important oxidative state [13], are overlapped in these extracts with those bands coming from the carbonyl groups existing in the antioxidants. Thus, signals are observed in this spectral zone even for the sample HMRec0 due to the presence of Irganox 1076, which contributes to the set of bands whose maximum appears at 1740 cm^−1^. As the content of HMDeg increases in the recycled HMRec material, the contribution of these bands associated with Irganox 1076 is lowered due to the slight decrease of its content, as seen in Fig. 2. Furthermore, they are fully masked at increasing amounts of HMDeg because their complete merging with the bands from oxidized species, with a total area that rises significantly in the films with the highest compositions in degraded HMDeg. These results are consistent with the ones obtained for the additive determination, in which consumption of Irgafos 168 was progressive and even complete when content in HMDeg was high. This could indicate that the amount of additives in the formulation is insufficient, and that there is still a large number of radical species that can induce premature degradation of these films when exposed to degrading environments, as will be seen later in this work.

**Fig. 4:**
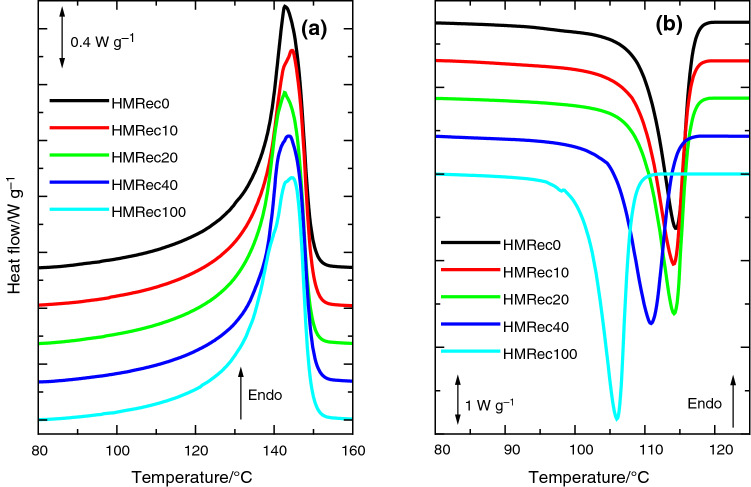
**a** First melting endotherms and **b** crystallization exotherms obtained from experiments performed at 20 °C min^−1^ for the different extruded recycled materials with different contents in HMDeg

Presence of these degradation species can alter phase transitions and crystalline structure if their amount is high enough, since those oxidative species contribute to interrupt chain regularity and could eventually lead to chain scission processes in the materials [13, 31], as deduced from variations in the MFR values (see results in Table 1). Those possible changes have been first analyzed by DSC. Figure 4a shows the similarity found in the melting curves obtained from the first heating process for all the recycled HMRec materials. The melting temperature, *T*_m_, is located at around 144 °C independently of content in HMDeg. Degree of crystallinity derived from this transition does not change much with composition in the recycled specimens. Its value is 0.58 for HMRec0, although a slight increase is noted in the recycled materials HMRec10 and HMRec20, reaching 0.62 and 0.61, respectively.

Results from the subsequent cooling experiments are much more interesting since the existing oxidative species could affect considerably the crystalline structure developed during the crystallization process. Thus, the cooling curves show an evident dependence on the HMDeg amount in the final recycled material, as deduced from Fig. 4b. The extruded HMRec0 displays a crystallization temperature, *T*_c_, of 114.5 °C, but location of this exotherm is moved to lower temperatures as HMDeg content increases, slightly first for HMRec10 or HMRec20 and more importantly in HMRec40 and extruded HMRec100, corroborating the effect of HMDeg presence in the ordering process [13]. This behavior clearly indicates a delay in the crystallization and, then, the beginning and completion of this process take place at lower temperatures. Nevertheless, the crystallinity degree reached is rather independent of the amount of HMDeg in the recycled HMRec material, and a value of 0.51 is derived from these exothermic processes.

Once differences have been observed in the crystallization process, being dependent on composition in HMDeg, knowledge on type of crystalline forms developed is mandatory since iPP shows a polymorphic behavior [32–37] and its properties strongly vary with the kind (and perfection) of crystallites. Three different polymorphic lattices, *α*, *β* and *γ*, all of them sharing a three-fold conformation, have been described in iPP, together with a phase of intermediate or mesomorphic order obtained by fast quenching [32, 34–38]. Additionally, a new crystalline form was reported more recently for iPP copolymers with 1-hexene or 1-pentene as counits at relatively high contents [39, 40]. Later, it was also observed in terpolymers including these two comonomers and even in terpolymers with 1-pentene and 1-heptene as counits [41, 42].

**Fig. 5:**
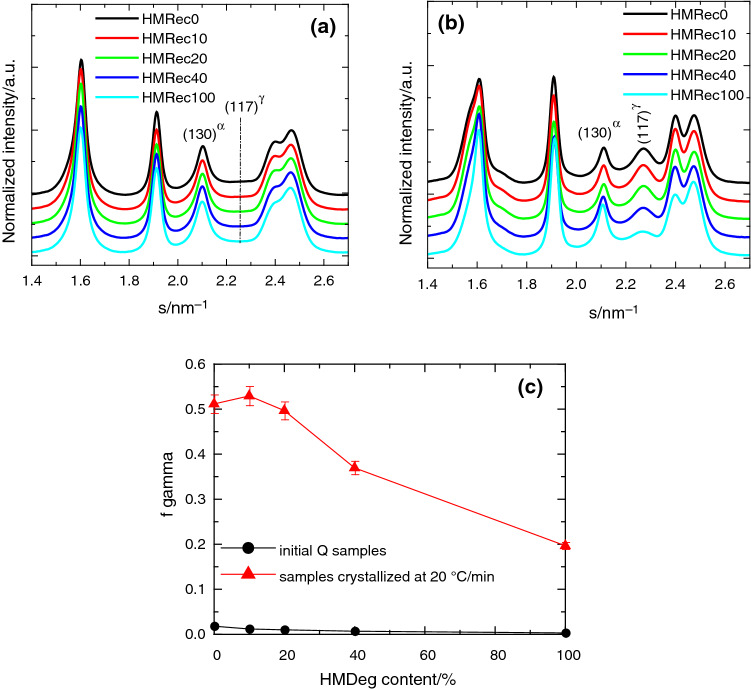
Synchrotron X-ray profiles at room temperature for: **a** the pristine iPP and the different recycled samples, and **b** neat iPP and HMRec materials after cooling at 20 °C min^−1^. **c** Relative content in the *γ* orthorhombic polymorph estimated from the different profiles: rapidly cooled (solid circle symbols) or after crystallization at 20 °C^.^min^−1^ (solid triangle symbols) specimens

Figure 5a shows the synchrotron X-ray profiles, at room temperature, for all the initial compression molded films attained from the extruded recycled HMRec materials after applying a fast cooling. Monoclinic form is noticed in all of them, independently of amount of the severely degraded HMDeg used in the recycling. This lattice is deduced by presence of their characteristic (110), (040), (130), (111), (131)/(041) and (060) crystalline planes. Degree of crystallinity can be achieved from these patterns and the values deduced are in agreement with those derived from first heating DSC experiments.

Situation is rather different if crystallization takes place at the conditions used for the cooling DSC experiments, i.e., at a rate of 20 °C min^−1^. Now, coexistence of monoclinic and orthorhombic modifications is noticeable in Fig. 5b, and its ratio is clearly dependent on HMDeg content in the recycled HMRec material. Presence of the *γ* orthorhombic lattice is deduced from observation of its (117) characteristic diffraction. Coexistence of these two crystalline forms is associated firstly with the metallocene origin of the iPP matrix evaluated and, secondly, with the lower rate applied during crystallization (in relation to the initial rapidly cooled specimens), since the orthorhombic modification is favored as rate is decreased [43].

Contribution of each type of crystalline lattice to the overall crystallinity can be determined from X-ray profiles represented in Figs. 5a, b by the ratio of areas of their respective characteristic reflections: the (130) one for monoclinic crystals, located at a value of *s* of 2.01 nm^−1^; and, the (117) diffraction placed at 2.25 nm^−1^, which is associated with the *γ* lattice [44, 45]. Figure 5c shows the completely different scenario for the orthorhombic polymorph as function of crystallization conditions: this crystalline form is almost completely absent in the quenched specimens just processed while a very significant amount is developed at a crystallization rate of 20 °C min^−1^, reaching values of more than 50% in some cases. It is also clearly noticed that formation of this γ form is hindered as content in HMDeg is increased. In fact, HMRec0 and HMRec10 specimens are able to develop similar amounts of orthorhombic lattice. Then, a reduction occurs, slight for the HMRec20 sample and more significant for the recycled HMRec40 and HMRec100 materials, so that only a 20% of global crystallinity in HMRec100 is *γ*-type. The behavior observed in HMRec100 is rather similar to that reported for HMdeg, where an important delay in the crystallization ability and a considerable decrease in the content of the orthorhombic form were described [13]. The additional incorporation of small amount in antioxidants prior to extrusion of the recycled samples seems not to affect the iPP crystallization capability in a considerable extent.

**Fig. 6:**
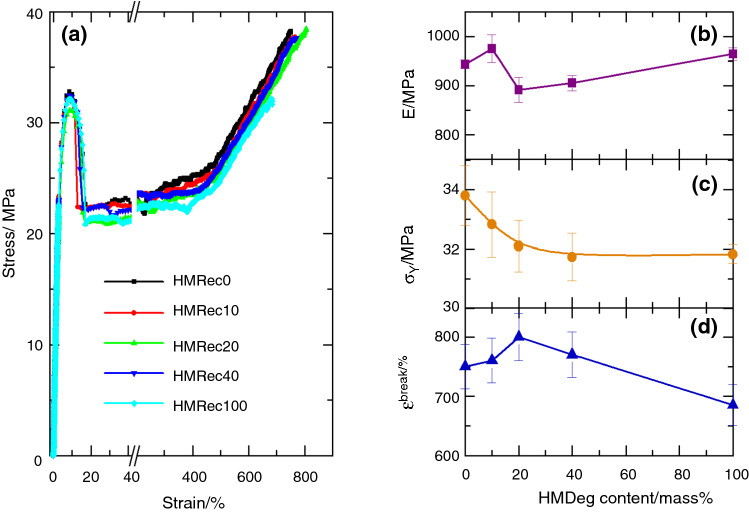
**a** Stress–strain curves for all the recycled materials. Average values of: **b** Young’s modulus; **c** yield stress; and, **d** strain at break

Those variations in the crystalline characteristics might induce changes on the mechanical behavior of these recycled HMRec materials. Accordingly, stress–strain tests have been carried out. Figure 6a shows the engineering stress–strain results for all the specimens at room temperature. These curves are characteristic of the standard cold drawing behavior of ductile polymers. Three different zones are then evidently noted in all of them: first, the elastic component, where the stress increases linearly with strain up to yielding point is reached. Thus, Young’s modulus can be estimated from the slope of curve in this part. The maximum point is considered as the minimal stress required for inducing a permanent deformation in the material. The second is the region where the stress is maintained relatively constant with increasing strain and it is related to the neck propagation. And, the last zone, designated as strain hardening, is distinguished by an important increase of stress with strain, this rise being associated with the stress-induced orientation of polymeric macrochains. This process ends with the break of the material, which is characterized by the tensile strength (*σ*_break_) and elongation at break (*ε*_break_).

Several mechanical parameters can be achieved from these curves, some of them being represented in Figs. 6b, c and d. A very remarkable and interesting feature can be deduced from all of them: mechanical response under tension at room temperature does not change significantly (variation is inferior to 10%) in these recycled HMRec materials, even in the HMRec100 when compared with that behavior shown by HMRec0.

Parameters related to stiffness might be expected to increase markedly as HMDeg content is raised because degradation shortens the length of the chain, minimizing entanglements. Furthermore, development of voids and crazes could be enhanced, which would add residual stresses [46, 47] and favor the increase of stiffness. Simultaneously, elongation capability should be considerably reduced. The results, however, do not show those detrimental effects, regardless the changes found in MFR values. In fact, Young’s modulus (*E*) increases slightly in HMRec10, it shows a minimum in HMRec20 and then it rises in HMRec40 and HMRec100, reaching a value even slightly higher than that for HMRec0. Regarding yield stress (*σ*_Y_), it slightly decreases in HMRec10 and HMRec20 reaching a plateau for HMRec40 and HMRec100. And, concerning the strain at break (Fig. 6d), it exhibits an opposite variation to that shown by E. Nevertheless, the changes in all of these parameters are almost within the experimental error. This striking behavior is associated with the action of incorporating antioxidants during the extrusion process, which are preventing the detrimental characteristics of degraded macrochains existing in HMDeg [13] from manifesting.

These results are analogous to those previously obtained by Martins et al. [48]. In another study where alternation of extrusion steps with aging stages was applied, it was observed that after extrusion the degree of crystallinity decreased while it increased after the aging stage [49], indicating a possible dilution of the degradation nuclei through the new films. This feature is also revealed in this work from the infrared results, which could trigger relative maintenance of mechanical behavior. The degradation points could be isolated by a proper action of antioxidants if they are well randomly distributed within the non-degraded polymeric chains.

**Fig. 7:**
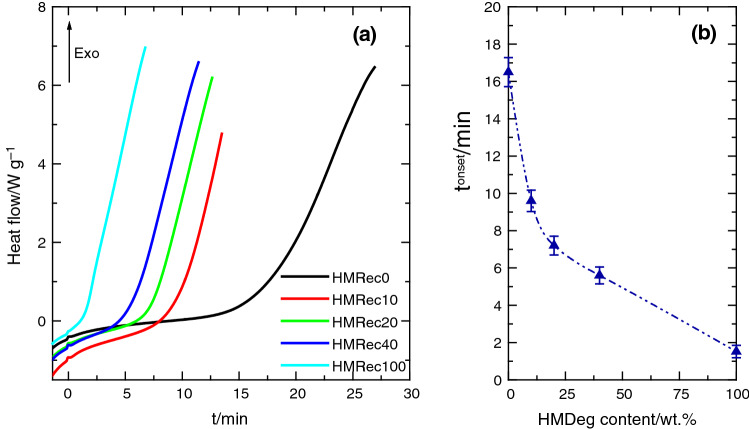
**a** Thermo-oxidative response under oxygen atmosphere at 170 °C for the different recycled HMRec materials. **b** Values for the onset of oxidation (*t*^onset^) in the several HMRec materials

In order to get a deeper insight on the effectiveness of antioxidants in the degradation state of these recycled HMRec materials, experiments of oxidation induction time have been performed [16, 50, 51]. It should be reminded at this point that a rather similar content of Irganox 1076 was found (see Fig. 2) in the specimens after extrusion for all the compositions in HMDeg. On the contrary, Fig. 7a shows great differences in the OIT curves depending on the HMDeg amount. This behavior seems to indicate the important effect that an increase in species capable of generating free radicals (which can catalyze degradation in contact with oxygen at temperatures above T_m_) plays on the time for the onset of oxidation (*t*^onset^) (see Fig. 7b), even though the content in antioxidants is practically constant. Thus, efficiency of antioxidants at high temperature is noticeably reduced and a content in HMDeg as low as 10 mass% in the recycled material implies an important reduction in the oxidation induction time. The greater amount of HMDeg is incorporated, the shorter time for the start of oxidation is observed, as shown in Fig. 7b, in such a way that immediately after changing the test atmosphere to oxygen an exothermic process appears in the HMRec100, which is the material with the highest content in oxidizing species.

Besides that particular behavior at very high oxidation temperature (170 °C), the important aspect with a greater practical significance is the durability of these recycled HMRec materials in the solid state. Resistance to aggressive conditions has been assessed in the current case as the time that these recycled HMRec films can remain at 95 °C without whitening and without undergoing an extreme fragility, as commented in the Experimental section. Thus, this accelerated aging test at 95 °C allows learning effectiveness of the mixture of antioxidants (750 ppm in Irgafos 168 + 300 ppm in Irganox 1076) used initially in these HMRec materials. These results will be compared to the ones exhibited by HMDeg [13], which was actually prepared in an accelerated test at 95 °C for 8 days. Nevertheless, it should be reminded, as aforementioned, that the extruded virgin HM films only contained 290 ppm of Irgafos 168 [13] before their exposure to the thermal oxidation treatment, that amount being much smaller than the content in antioxidants for some of the present recycled HMRec materials, as deduced from Fig. 2.

**Fig. 8:**
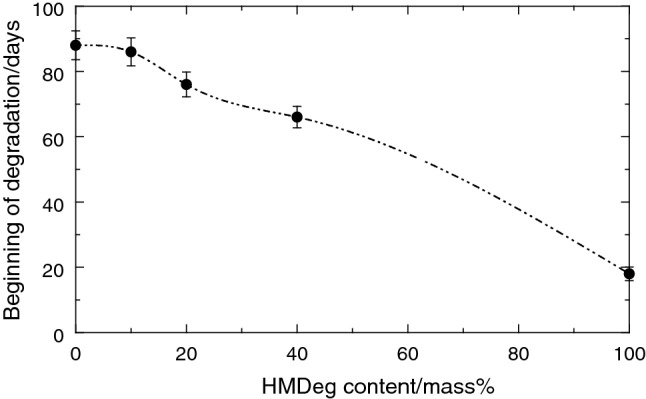
Resistance to thermal-oxidation for the different recycled HMRec materials exposed to a degradation test at 95 °C

Figure 8 shows the time that the different extruded recycled HMRec films are able to stay imperturbable in the convection oven. As expected, this time is dependent on the HMDeg content incorporated into the recycled material since remaining amount in antioxidants decreases as HMDeg rises (see Fig. 2). In fact, the extruded HMRec0 specimen, containing 560 ppm in Irgafos 168 and 270 ppm in Irganox 1076 before beginning of the accelerating test, reveals no whitening or brittleness indications up to 88 days elapsed, time much higher than the 8 days observed in the initial HM sample, which led to HMDeg.

A considerably lower content in Irgafos 168 remains in the recycled HMRec10 sample (100 ppm in Irgafos 168 and 210 ppm in Irganox 1076) compared to the HMRec0 specimen. This indicates that consumption during its manufacture by extrusion has been higher in the former. Thus, need of higher amounts of antioxidants is anticipated for the processing stage in order to deactivate the radical species present in the severely degraded HMDeg material used as recycling component. Content in Irganox 1076 is, however, only an 18% inferior in HMRec10 regarding that in HMRec0. This fact remarks the importance of presence in Irganox 1076, which is a phenolic antioxidant, in the protection of this recycled HMRec10 material under the conditions of the aging test, as deduced from its similar degradation time, 86 days, seen in Fig. 8.

A gradual reduction in the beginning of degradation (whitening and appearance of fragility) is observed in Fig. 8 for the other recycled materials with higher HMDeg content, although their Irganox 1076 amount is rather analogous to that found in HMRec10 (see Fig. 2). This fact can be associated with initiation of an imbalance between action of antioxidants, increasing amount in oxidative species and variations in the MFR values as content in HMDeg is raised. But resistance to thermo-oxidative treatment was superior to that exhibited by the material that only contained Irgafos 168 [13] even in the extruded HMRec100 specimen, with 18 days for the beginning of degradation, compared with the no more than 8 days for the original HMDeg.

**Fig. 9:**
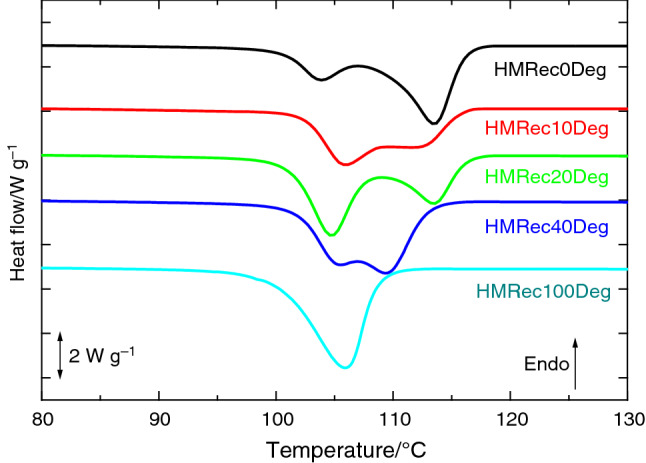
DSC cooling curves at 20 °C min^−1^ performed to the different recycled HMRec materials after their exposure to a thermo-oxidation treatment at 95 °C

In addition to appearance of whitening and fragility, the state of severe degradation of the different recycled materials exposed to the thermo-oxidation test at 95 °C was confirmed by means of a cooling DSC run, represented in Fig. 9, through the displacement of their *T*_c_ to lower temperatures, which was indicative of the characteristic crystallization delay found in a severely degraded HM specimen, i.e., in HMDeg [13].

Recycled HMRec materials with up to a 40 mass% in HMDeg display analogous DSC cooling curves that the curves described in literature [13], where a splitting of the crystallization process took place because of their state of degradation. Sample HMRec100Deg, however, displays a single peak, at 106 °C, which is coincident with the lower temperature component of the other degraded HMRec materials (HMRec0Deg, HMRec10Deg, HMRec20Deg and HMRec40Deg) since its degradation is even more severe.

Summarizing, the excellent mechanical response of the recycled materials is mainly associated with a proper action of the antioxidants during melt extrusion, resulting on a rather optimal durability of these recycled HMRec materials in the solid state under aggressive conditions.

## Conclusions

Recycled model materials have been prepared by extrusion from virgin metallocene iPP (HM) with varying amounts of severely degraded HMDeg (attained from that iPP subject to an accelerated aging test at 95 °C) and a known amount of two antioxidants. Consumption of these antioxidants is strongly dependent on amount of degraded HMDeg incorporated. The antioxidant species most drastically reduced correspond to Irgafos 168, while Irganox 1076 undergoes only a slight reduction during the preparation process by extrusion. This fact can be associated with the higher content of oxidative radicals existing in the macromolecular chains of the severely degraded HMDeg polypropylene.

Crystallization capacity of recycled HMRec materials is also determined by the amount of HMDeg. Thus, the crystallization exotherm is moved to lower temperatures as HMDeg content increases, indicating a delay in the crystallization, although the crystallinity degree reached is rather independent of the amount of HMDeg in the recycled HMRec material. Furthermore, orthorhombic development is hindered in the recycled materials with the highest compositions in HMDeg.

On the other hand, mechanical response does not practically change (variation is inferior to 10%) in these recycled HMRec materials compared with that shown by HMRec0, even in the HMRec100 specimen. This excellent behavior is associated with a dilution effect of the radicals present (mainly in HMDeg chains) together with a proper action of the antioxidants during melt extrusion.

Presence of higher amounts in antioxidants during a durability test at 95 °C in these recycled materials postpones the beginning and progress of oxidation processes at high temperature, but once these additives are consumed, similar degradation characteristics (whitening and extreme fragility) are observed in these metallocene iPP-based materials.

The main conclusion is, therefore, the excellent mechanical behavior observed under tension in the recycled materials, which is mainly associated with a proper action of the antioxidants during melt extrusion, resulting on a rather optimal durability of these recycled HMRec materials in the solid state under aggressive conditions. Thus, an appropriate antioxidants formulation is mandatory for polyolefins, in general, and, more importantly, for their recycling in order to achieve a tailored and controlled protection at high temperatures and during their service life. With those formulations, a suitable performance of more sustainable materials can be reached.
